# A Dual-Band Patch Antenna with Combined Self-Decoupling and Filtering Properties and Its Application in Dual/Squad-Band Two-Element MIMO Array

**DOI:** 10.3390/s24216833

**Published:** 2024-10-24

**Authors:** Jun-Yi Lv, Jun-Ming Zhang, Peng-Fei Lv, Li-Xin Xu

**Affiliations:** 1 Shenzhen Institute for Advanced Study, University of Electronic Science and Technology of China, Shenzhen 518110, China; junyi.lv@std.uestc.edu.cn; 2 School of Electronic Science and Engineering, Beijing University of Posts and Telecommunications, Beijing 100000, China; 2022110341@bupt.edu.cn; 3 Ocean College, Jiangsu University of Science and Technology, Zhenjiang 212003, China; lxu@just.edu.cn; 4 Jiangsu Marine Technology Innovation Center, Nantong 226199, China

**Keywords:** dual-band patch antenna, filtering antenna, radiation null, low mutual coupling, multi-band multiple-input multiple-output (MIMO) array

## Abstract

This paper proposes a dual-band patch antenna with combined self-decoupling and filtering properties, designed to suppress mutual coupling between two antenna elements both within the same dual-band and across different dual-bands. Initially, a dual-band aperture-coupled filtering patch antenna is designed, featuring a forked short-circuited SIR feedline with a quarter-wavelength open-ended stub and a U-shaped patch with two U-slots, which generate three controllable radiation nulls while introducing two additional resonant modes. The design steps are also provided in detail. Subsequently, the low mutual coupling phenomenon of two vertically placed aperture-coupled patch antennas is investigated, successfully developing a high-isolated dual-band two-element MIMO array I. Furthermore, the other quad-band two-element MIMO array II is designed, which utilizes the filtering response to significantly reduce mutual coupling across four bands. Finally, a dual-band filtering patch antenna element and two two-element MIMO arrays are fabricated and measured. The measurements and simulations validate the antenna’s low mutual coupling performance in multi-band MIMO arrays and demonstrate its strong potential for future wireless communication applications.

## 1. Introduction

The advent of the 5G era demands communication systems with high transmission rates and large data-transfer capacities; more and more microwave devices that support multiple bands and multi-functionality are being adopted. Among these, dual-band filtering antennas and multi-band MIMO arrays with isolation enhancement have become hot spots.

Among the various antenna options, patch antennas are particularly promising due to their simple design, cost-effectiveness and ease of manufacturing. Traditionally, utilizing patch radiator as a resistor–inductor–capacitor (RLC) resonator to replace the last stage of a filter network, a filtering patch antenna is designed based on filter synthesis [1,2,3,4,5], which results in high integration. This method, however, complicates the feed network and introduces additional insertion loss. Therefore, developing filtering patch antennas without extra filtering circuits is essential. For instance, a filtering property can be achieved using half-wavelength strips [6], folded T-shaped strip [7], shorting pins [8], shared stepped impedance resonators (SSIRs) and complementary split-ring resonators (CSRRs) [9]. These antennas possess good filtering properties. However, these filtering patch antennas are applied in single-band scenarios. Consequently, researchers have suggested various methods to develop dual-band filtering patch antennas [10,11,12,13,14]. In [10], stepped impedance resonators (SIRs) are integrated directly into the antenna structure based on filter theory, successfully achieving a dual-band filtering antenna. However, the peak gain is relatively low, with measured gain of −1.8 and 1.1 dBi in the two passbands. Dual-mode T-type resonators are integrated with two vertically placed patch antennas, where the T-resonator generates three transmission zeros [11]. Furthermore, dual-band filtering antennas are achieved by integrating U-slots [12,13] and stacking parasitic patches [14]. However, stacking multiple parasitic patches significantly increases the antenna’s height.

MIMO technology can simplify resource allocation and effectively enhance system capacity [15]. However, mutual coupling between antennas operating at the single- or multi-band in limited space significantly impacts system performance, facilitating the emergence of various decoupling methods. On the one hand, common methods for achieving single-band decoupling include decoupling networks [16,17], array decoupling surfaces [18], mode cancellation [19,20], energy cancellation [21,22], slot loading [23] and combination of a decoupling network and structures [24]. Mutual coupling is mitigated by introducing an additional path with the same amplitude and opposite phase in the passband [16,17,18]. In [21], high isolation is achieved by adjusting the ratio of electric and magnetic coupling through etching different regions on the non-radiating edge. In [24], both the decoupling feeding network and structures are adopted to obtain two decoupling nulls to reduce mutual coupling; the minimum isolation enhancement greater than 30 dB exists within 2.3–2.5 GHz. Although they are effective for decoupling within the single band, achieving multi-band decoupling is challenging. On the other hand, without adding any decoupling elements, filtering antenna elements for single-band operation have been shown to achieve low mutual coupling across two different frequency bands [25,26]. However, when applied to MIMO arrays operating in four bands, four antenna elements are required, which results in occupying more space. In [27], self-decoupling between dual-band patch antennas is realized using hybrid coupling interfaces. Nonetheless, achieving decoupling across four bands poses considerable design difficulties.

So, how to simply and effectively suppress mutual coupling in a MIMO array operating in dual-band and quad-band remains a significant challenge. To address the issues, this paper proposes a dual-band patch antenna with integrated self-decoupling and filtering properties, along with detailed design steps. Simulations and experiments confirm that the proposed antenna element effectively improves isolation in dual-band and quad-band two-element MIMO arrays. The main contributions of this paper are summarized as follows:

1. We develop a dual-band filtering antenna element specifically for MIMO arrays, thoroughly investigating its working mechanism and the impact of key parameters on performance, while providing a detailed design methodology.
2. We explore the inherent decoupling characteristics of aperture coupling, proposing a design that achieves high isolation and filtering response in the dual-band two-element MIMO array I.
3. We design two different dual-band filtering antennas for elements of the quad-band two-element MIMO array II. We improve the isolation across the quad-band using the filtering characteristics of the antenna elements.
4. We validate the effectiveness of the proposed method through comparisons of measured and simulated results. The consistent outcomes indicate that the developed dual-band filtering antenna element has promising applications in multi-band multi-antenna systems.

The rest of this paper is organized as follows:

In Section 2, we elucidate the working mechanism of the dual-band filtering antenna element. In Section 3, we investigate two two-element MIMO arrays with high isolation. In Section 4, we complete the fabrication, assembly and testing of a dual-band filtering antenna element and two two-element MIMO arrays, comparing the results with related works. Finally, in Section 5, we summarize the paper and outline future research directions.

## 2. Dual-Band Filtering Antenna Element Design and Analysis

In this section, we delve into the working mechanism of the dual-band filtering antenna element by analyzing the current distribution and explore the impact of key parameters on antenna performance, thereby providing guidance for the design of this antenna element.

### 2.1. Antenna Element Structure

**Figure 1:**
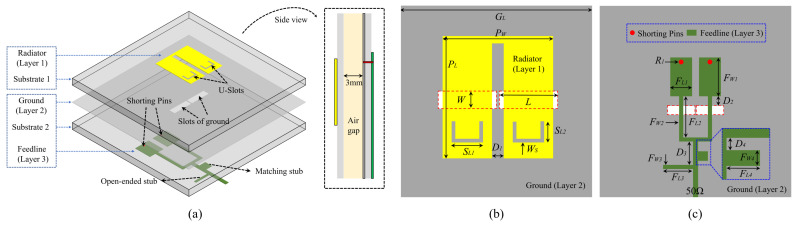
The structure of the proposed dual-band filtering antenna element: (**a**) three-dimensional and side views. (**b**) Top view (Layer 1 and Layer 2). (**c**) Bottom view (Layer 2 and Layer 3).

As depicted in Figure 1a, the antenna element introduced in this study employs a dual-layer dielectric substrate design. Each substrate is 1 mm thick, fabricated from FR4 material, featuring a dielectric constant of 4.4 and a loss tangent of 0.02. These two dielectric substrates are separated by a 3 mm air gap. The top of Substrate 1 prints Layer 1, while Layers 2 and 3 are printed on the top and bottom of Substrate 2, respectively. Figure 1b illustrates the top view, displaying Layers 1 and 2, where Layer 1 consists of a U-shaped patch with two U-slots and Layer 2 acts as the ground plane etched two rectangular slots. Figure 1c shows the bottom view, detailing the arrangement of Layers 2 and 3. The feedline in Layer 3 features a forked short-circuited SIR structure, accompanied by an open-ended stub and a matching stub. Signal transmission to the antenna element is facilitated by a 50 Ω SMA connector. Comprehensive dimensions of antenna element 1 are listed in Table 1.

### 2.2. Working Mechanism

**Figure 2:**
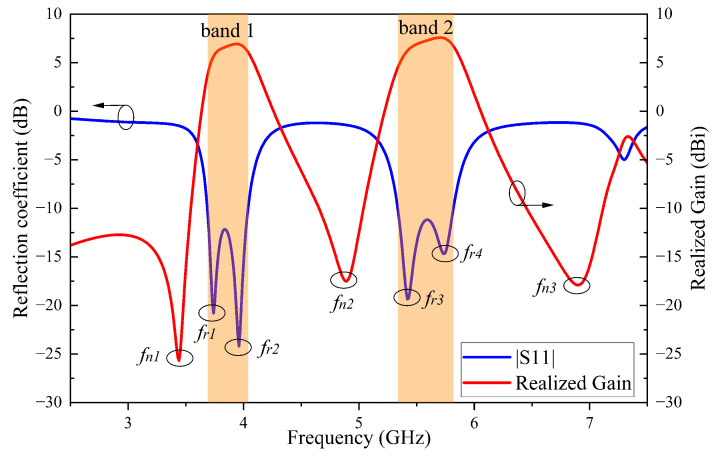
Simulated reflection coefficient and realized gain of the proposed antenna element.

Figure 2 presents the simulated S-parameters and realized gain of the proposed antenna element 1. The results clearly indicate a dual-band operation within the −10 dB impedance bandwidth. The lower band spans from 3.69 to 4.04 GHz (part of the n77 band), while the higher band spans from 5.34 to 5.82 GHz (part of the 5G WiFi band). Each band contains two resonant frequencies, designated as $f_{r1}$, $f_{r2}$, $f_{r3}$ and $f_{r4}$. Furthermore, the introduction of three radiation nulls ($f_{n1}$, $f_{n2}$ and $f_{n3}$) in the stopband sharpen roll-off at the dual-band edges. Notably, these nulls can be independently tuned. A detailed current distribution analysis is performed to further understand the antenna’s working mechanisms, focusing on the resonant modes and radiation nulls.

#### 2.2.1. Analysis of Resonant Modes and Radiation Nulls

**Figure 3:**
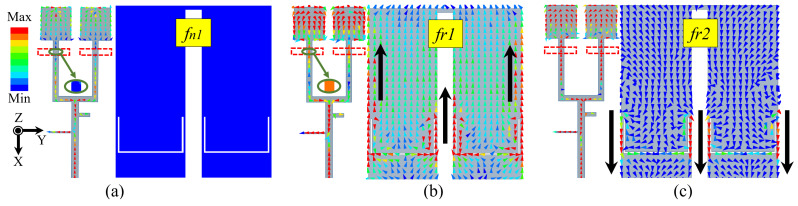
Surface current distributions on patch and feedline at (**a**) *fn1* = 3.44, (**b**) *fr1* = 3.74 GHz and (**c**) *fr2* = 3.96 GHz.

The aperture-coupled patch antenna utilizes the short-circuited SIR feedline to generate a radiation null ($f_{n1}$) and introduce an additional resonance mode ($f_{r1}$) [28]. Figure 3a,b show the current distribution on the feedline and patch at $f_{n1}$ and $f_{r1}$. At $f_{n1}$, the short-circuited SIR structure resonates and the current amplitude at the feedline’s coupling position is nearly zero, preventing energy coupling to the patch, thereby resulting in a radiation null $f_{n1}$. At $f_{r1}$, the feedline allows strong energy coupling to the patch, where the primary current flows in the −X direction. Figure 3c shows the current of TM10 mode influenced by the U-slots at $f_{r2}$, with the primary current flowing in the +X direction.

**Figure 4:**
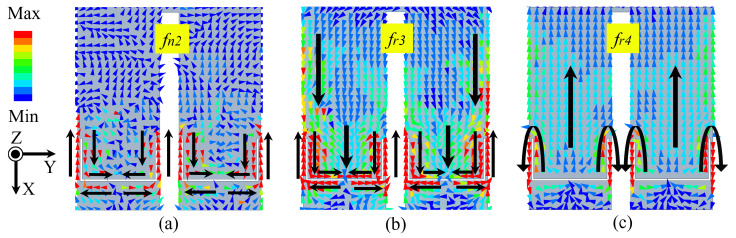
Surface current distributions on patch at (**a**) *fn2* = 4.89, (**b**) *fr3* = 5.43 GHz and (**c**) *fr4* = 5.74 GHz.

Figure 4a,b show the current distribution on the patch at $f_{n2}$ and $f_{r3}$. The results indicate that the current is mainly concentrated around the U-slots. At $f_{n2}$, the U-shaped slot resonates on the patch and the current is out of phase, resulting in mutual cancellation of far-field radiation. The total length of U-slots is half of the guiding wavelength in FR4 material at $f_{n2}$. At $f_{r3}$, the overall currents of patch flow in the +X direction. Thus, the introduction of resonance frequency $f_{r3}$ and radiation null $f_{n2}$ can be attributed to the U-slots etched on the patch. Figure 4c shows the current of TM20 mode influenced by the U-slots at $f_{r4}$. Typically, the TM20 mode exhibits low axial radiation efficiency due to the out-of-phase current distribution. However, in this case, the currents beneath the patch are reshaped by the U-slots, causing the overall currents to flow in the +X direction, thereby improving axial radiation.

**Figure 5:**
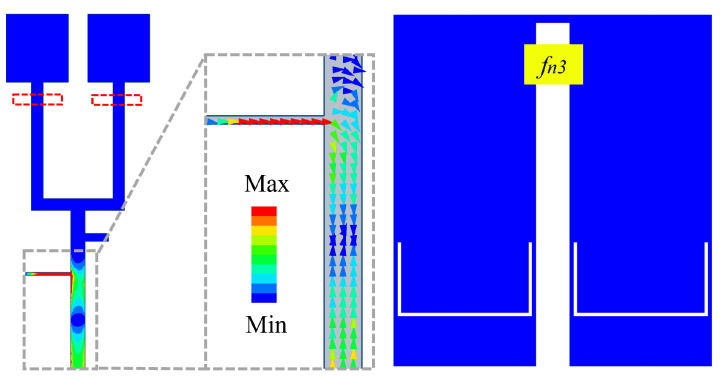
Surface current distributions on patch and feedline at *fn3* = 6.9 GHz.

To further investigate how the third radiation null ($f_{n3}$) is generated, Figure 5 shows the current distribution on the feedline and patch at fn3. The results indicate that at fn3, the current is primarily concentrated on the open-ended stub, which acts as a quarter-wavelength resonator [12], its total length is a quarter of the guided wavelength in FR4 material at $f_{n3}$. Consequently, the open-ended stub functions as a resonant filter at $f_{n3}$, preventing the signal from flowing along the feedline and thus not exciting the patch, introducing the third radiation null ($f_{n3}$).

#### 2.2.2. Parameter Study

Examining several important parameters in detail enhances our understanding of the working mechanisms of the proposed dual-band filtering antenna element 1 with three controllable radiation nulls.

**Figure 6:**
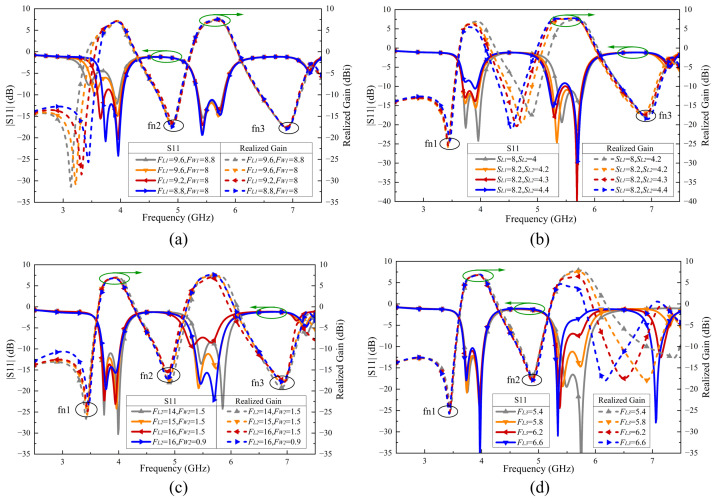
Performance of the proposed filtering antenna element 1 with different parameters. (**a**) $F_{L1}$ and $F_{W1}$. (**b**) $S_{L1}$ and $S_{L2}$. (**c**) $F_{L2}$ and $F_{W2}$. (**d**) $F_{L3}$.

Figure 6a illustrates how varying the lengths ($F_{L1}$) and widths ($F_{W1}$) of short-circuited SIR structures affects the performance of antenna element 1. As the lengths ($F_{L1}$) and widths ($F_{W1}$) of the short-circuited SIR structures increase, the $f_{r1}$ along with $f_{n1}$ move to lower frequencies. This indicates that the short-circuited SIR structures play a crucial role in controlling $f_{r1}$ and $f_{n1}$. By adjusting ($F_{L1}$) and ($F_{W1}$), we can independently tune fr1 and fn1. The influence of the lengths ($S_{L1}$ and $S_{L2}$) of the U-slots in the radiating patch is also investigated as shown in Figure 6b. As the U-slots lengthen, $f_{r3}$ and $f_{n2}$ move lower, simultaneously affecting the impedance matching of the lower band. We can alter the total length of the U-slots to adjust the $f_{r3}$ and $f_{n2}$. Subsequently, Figure 6c illustrates the influence of the forked section ($F_{L2}$ and $F_{W2}$). Increasing $F_{L2}$ and $F_{W2}$ results in a downward shift of the resonant frequencies $f_{r3}$ and $f_{r4}$, while significantly influencing the impedance matching of the higher band. Finally, Figure 6d outlines the impact of loading open-ended stub. As the open-ended stub lengthens, only $f_{n3}$ shifts lower. Simultaneously, it severely influences the impedance matching of $f_{r4}$. We can independently control the position of $f_{n3}$ by varying $F_{L3}$.

**Figure 7:**
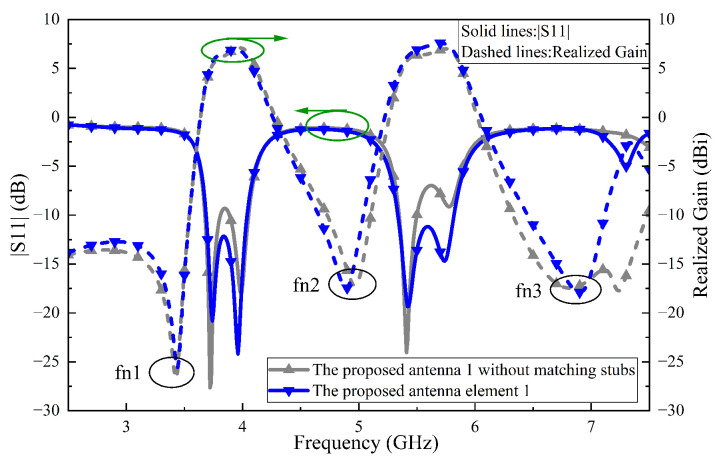
Simulated reflection coefficient and realized gain without and with matching stub.

Figure 7 compares the simulated reflection coefficients and realized gain of the antenna element 1 without and with the matching stub. The results indicate that the matching stub significantly improves the impedance matching in the dual-band.

#### 2.2.3. Design Guideline

Based on the above analysis, the following are the detailed steps for designing a dual-band filtering antenna with three controllable radiation nulls:

1. Employ an aperture-coupled patch along with a short-circuited SIR feedline to achieve an additional resonant mode $f_{r1}$ and the first radiation null $f_{n1}$.
2. Load a half-wavelength U-slot to introduce another resonant frequency $f_{r3}$ and the second radiation null $f_{n2}$.
3. Develop a forked, short-circuited SIR feedline to adjust the frequency of $f_{r3}$ and $f_{r4}$, optimizing the impedance of the higher band.
4. Add a quarter-wavelength open-ended stub to generate the third radiation null $f_{n3}$, forming dual-bandpass filtering characteristics.
5. Configure a matching stub and carefully adjust important parameters to ensure good impedance matching and sharpened roll-off rate for the dual-bands.

## 3. Design and Analysis of Two-Element MIMO Arrays with Enhanced Isolation

In this section, the dual-band filtering antenna under investigation is utilized as an array element in two distinct MIMO configurations. One is the two-element MIMO array I operating in the same dual-band and the other is the two-element MIMO array II operating across different dual-bands. Additionally, we will delve into the working mechanisms for achieving high isolation.

### 3.1. Two-Element MIMO Array I Operating in the Same Dual-Band

#### 3.1.1. The Working Mechanism for Enhancing Isolation

Research indicates that microstrip-to-slotline transitions are highly effective in suppressing common-mode signals [29,30]. When differential-mode signals propagate, the slotline’s central plane acts as a virtual electric wall, facilitating the transfer of the electric field from the microstrip to the slotline. This occurs because differential signals generate opposing currents on either side of the slotline, forming an effective transmission path. In contrast, during common-mode signal propagation, the slotline’s central plane serves as a virtual magnetic wall, resulting in the cancellation of common-mode signals within the slotline due to minimal vertical electric field penetration. In this paper, we leverage this mechanism to enhance the isolation between tightly vertically arranged aperture-coupled antenna elements.

**Figure 8:**
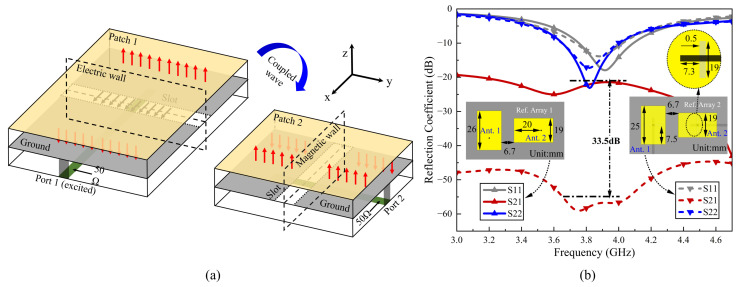
(**a**) Three-dimensional view of two vertically positioned aperture-coupled antennas. (**b**) Simulated S-parameters of Ref. Array 1 and Ref. Array 2.

Figure 8a demonstrates that when port 1 is excited, energy is transferred via the microstrip-to-slotline transition to patch 1, effectively exciting the TM10 mode. The electric field at the non-radiating edge of patch 1 along the *x*-direction displays a half-wavelength standing wave pattern, with peak amplitude at the ends and a minimum at the center, while the field amplitude and direction in the *y*-direction remain nearly uniform. Patch 2 is excited through coupled wave and exhibits a similar electric field distribution to patch 1 [17,20]. The electric field distribution of patch 2 in the y-direction reflects common-mode signal characteristics, with the slotline’s central plane acting as a virtual magnetic wall, thereby preventing energy transfer to port 2, thus achieving high isolation.

To clearly illustrate the decoupling effect, we compared the aperture-coupled two-element Ref. array 2 with a traditional coax-fed two-element Ref. array 1. As depicted in Figure 8b, the edge-to-edge distances of the two Ref. arrays’ elements are both 6.7 mm. The |S21| of Ref. array 2 is less than 54.8 dB throughout the entire passband and compared to Ref. array 1, isolation is improved by at least 33.5 dB. This simulated results indicate that almost no energy reaches port 2, providing strong validation of our above analysis. It should be noted that this decoupling mechanism diverges from parasitic slotlines between adjacent antennas, which depend on the band-reject response of slotlines [23].

#### 3.1.2. The Two-Element MIMO Array I with Enhanced Isolation

**Figure 9:**
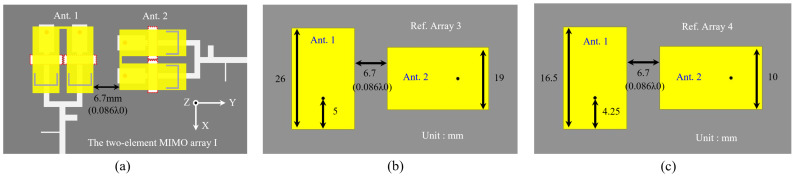
Top views of (**a**) the two-element MIMO array I, (**b**) Ref. Array 3 and (**c**) Ref. Array 4.

Figure 9 shows the top views of the two-element MIMO array I, Ref. array 3 and Ref. array 4, where the edge-to-edge distances of the units are set to 0.086 λ0 (6.7 mm), where λ0 is the free space wavelength at 3.87 GHz. The unit of the two-element MIMO array I is the dual-band filtering antenna element 1 introduced in Section 2. The units of Ref. arrays 3 and 4 are coax-fed patch antennas operating in the lower and higher bands, respectively.

**Figure 10:**
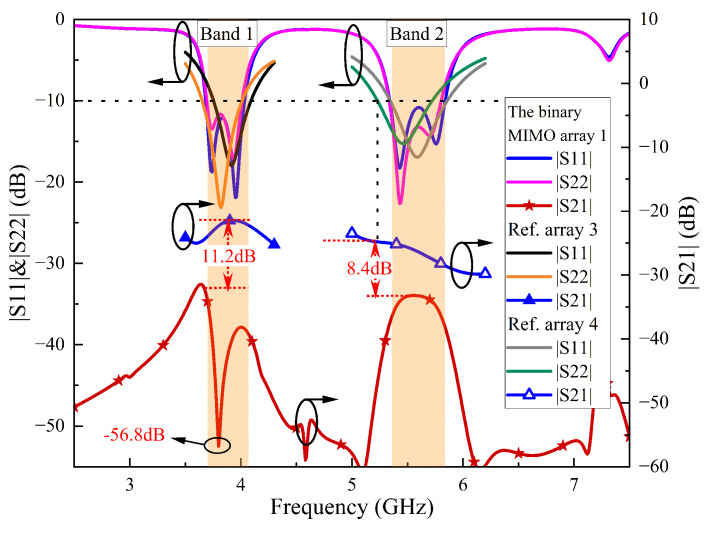
Simulated S-parameters of the two-element MIMO array I, Ref. Array 3 and Ref. Array 4.

Figure 10 shows the simulated S-parameters for the two-element MIMO array I, as well as Ref. arrays 3 and 4. Ant. 1 of the two-element MIMO array I operates in the 3.68–4.03 GHz and 5.34–5.84 GHz bands, while Ant. 2 operates in the 3.69–4.01 GHz and 5.34–5.81 GHz bands. The isolation is less than −32.8 dB in the two lower bands and less than −33.2 dB in the two higher bands. Ant. 1 of Ref. array 3 operates in the 3.76–4.09 GHz and Ant. 2 operates in the 3.66–4.01 GHz band, with isolation less than −21.6 dB in both bands. Ant. 1 of Ref. array 4 operates in the 5.35–5.85 GHz band and Ant. 2 operates in the 5.23–5.72 GHz band, with isolation less than −24.8 dB in both bands. As a result, the isolation of the two-element MIMO array I is improved by at least 11.2 (32.8–21.6) dB in the lower band and 8.4 (33.2–24.8) dB in the higher band compared to Ref. arrays 3 and 4. Additionally, The |S11| of Ref. array 3 and 4 elements exhibits significant frequency band shifts.

**Figure 11:**
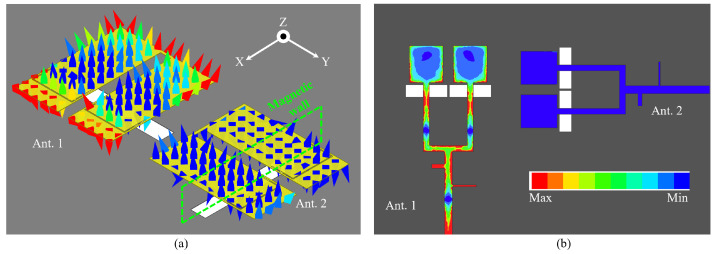
Electric filed distribution on (**a**) patches and (**b**) feedlines of two-element MIMO array I at 3.8 GHz.

Notably, a decoupling null occurs at 3.8 GHz in the lower band, achieving a maximum isolation of −56.8 dB, which is a maximum improvement of 34.2 (56.8–21.6) dB. Figure 11a shows the electric field distribution of the two patches in two-element MIMO array I when port 1 is excited. Consistent with the analysis in Figure 8a, the electric field distribution in the coupled Ant. 2 exhibits common-mode signal characteristics in the y-direction, with the central plane of the slots on the ground acting as a virtual magnetic wall to prevent energy transmission. Figure 11b illustrates the electric amplitudes on two feedlines, visually indicating that there is no energy flow at port 2. Additionally, the currents of resonant modes 3 and 4 of patch 1 are primarily coupled through the U-slots, resulting in unequal electric field magnitudes and directions in the y-direction. Consequently, the coupled Ant. 2 does not exhibit common-mode signal characteristics in the y-direction, leading to the absence of a decoupling null in the higher band. However, an enhanced isolation is still observed when compared to Ref. array 4.

**Figure 12:**
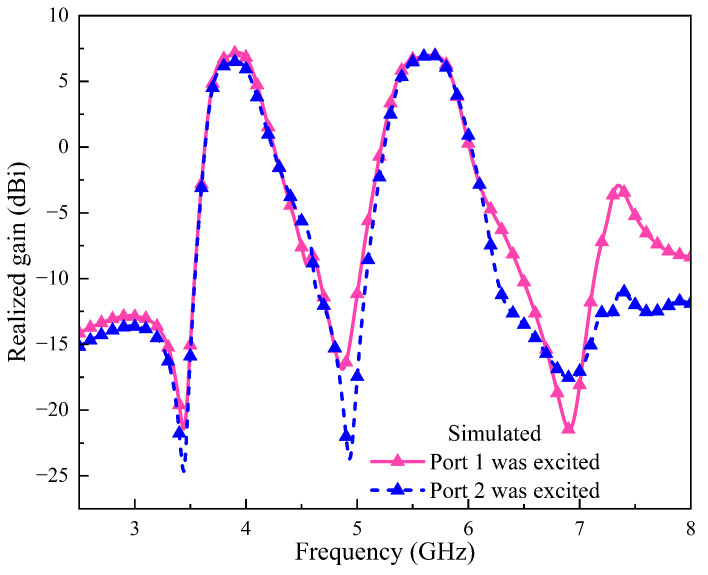
Simulated realized gain of the two-element MIMO array I.

Figure 12 shows the realized gain of the two-element array I. From the simulated curves, Ant. 1 achieves three radiation nulls at 3.43 GHz, 4.87 GHz and 6.91 GHz. Ant. 2 achieves three radiation nulls at 3.43 GHz, 4.94 GHz and 6.92 GHz. Both antenna elements exhibit a significant filtering response.

### 3.2. Two-Element MIMO Array II Operating Across Different Dual-Bands

#### 3.2.1. The Two-Element MIMO Array II Structure

**Figure 13:**
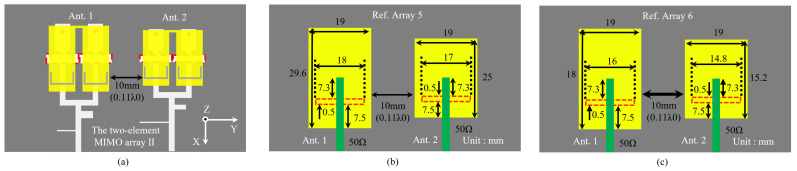
Top views of (**a**) the two-element MIMO array II, (**b**) Ref. Array 5 and (**c**) Ref. Array 6.

To achieve quad-band operation, two antenna elements that operate at different dual-bands are placed side by side, forming the two-element MIMO array II, as illustrated in Figure 13a. The edge-to-edge distance between Ant. 1 and Ant. 2 is set to 10 mm (approximately 0.11 λ0), where λ0 represents the free-space wavelength at 3.3 GHz. The specific dimensions of Ant. 2 are listed in Table 1. Following the design guidelines outlined in Section 2.2.3, another dual-band filtering Ant. 1 is designed to operate within the 3.17–3.47 GHz band and the 4.81–5.16 GHz band, with its detailed dimensions provided in Table 2. Specially, the lower bands of both antennas are adjacent, as are the higher bands. To fully evaluate the isolation improvement, two non-filtering aperture-coupled antenna arrays with the same edge-to-edge distance are introduced, named Ref. array 5 and 6, with specific dimensions shown in Figure 13b,c.

#### 3.2.2. The Two-Element MIMO Array II with Enhanced Isolation

**Figure 14:**
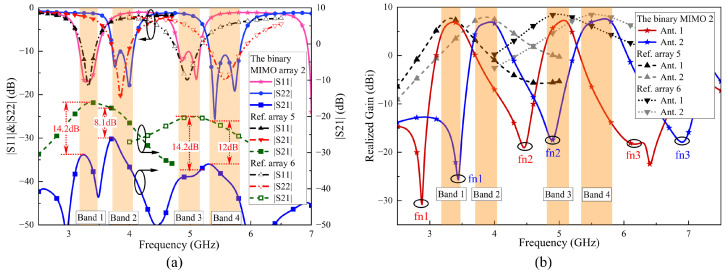
Simulated (**a**) S-parameters and (**b**) realized gain of two-element MIMO array II, Ref. array 5 and Ref. array 6.

Figure 14a presents the simulated S-parameters of the two-element MIMO array II, Ref. array 5 and 6. Ref. array 5 operates in the 3.22–3.42 GHz and 3.73–3.98 GHz bands, with simulated |S21| values of less than −16.2 and −17.9 dB, respectively. Ref. array 6 operates in the 4.78–5.13 GHz and 5.33–5.85 GHz bands, with corresponding |S21| values of less than −20.1 and −21.2 dB. At the same time, the |S21| values of the two-element MIMO array II in its four operating bands are less than −30.6, −26, −34.3 and −33.2 dB, improving by 14.4, 8.1, 14.2 and 12 dB compared to Ref. array 5 and 6.

Figure 14b illustrates the simulated realized gain of the two-element MIMO array II, Ref. array 5 and 6. The two-element MIMO array II demonstrates excellent in-band realized gain and filtering response. The radiation nulls $f_{n1}$ (3.47 GHz) and $f_{n2}$ (4.91 GHz) of Ant. 2 are located within the two operating bands of Ant. 1. Although the radiation nulls of element 1 do not precisely cover the operating bands of element 2, their presence still significantly improves the roll-off rate at the passband edges. In contrast, the realized gain of Ref. arrays 5 and 6 exhibits a smooth gradient at the passband edges. Through comparison, it is verified that the use of filtering antenna elements significantly improves isolation between antennas.

## 4. Measurement Verification and Comparison

In this section, we fabricate and test the filtering antenna element proposed in Section 4.1 and the two two-element MIMO arrays introduced in Section 4.2 and Section 4.3, comparing them with relevant studies. Here, S-parameters were measured by an Agilent N8722ES network analyzer; realized gains and radiation patterns were measured with a near-field Satimo StarLab system.

### 4.1. Antenna Element 1 Results and Comparison

#### 4.1.1. Fabricated Prototype of Antenna Element 1

**Figure 15:**
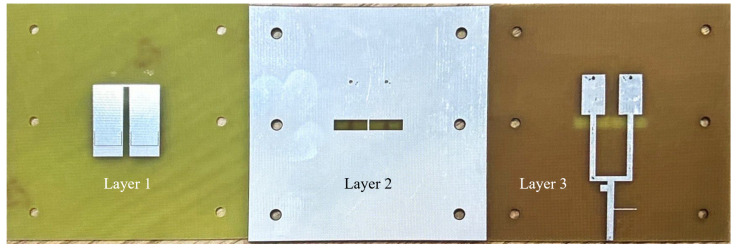
Fabricated prototype of the antenna element 1 described in detail in Table 1.

Figure 15 shows the fabricated prototype of Antenna Element 1 described in detail in Table 1. The six holes on the three layers facilitate the insertion of nylon pillars to support the two FR4 dielectric substrates.

#### 4.1.2. S-Parameters and Realized Gain Analysis

**Figure 16:**
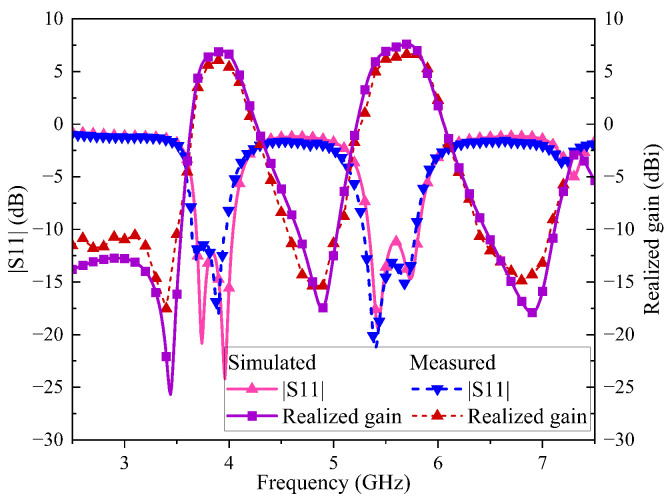
Simulated and Measured S-parameters and realized gain.

Figure 16 compares the measured S-parameters and realized gain with the simulated results. The measured −10 dB impedance bandwidths are 9.17% from 3.64 to 3.99 GHz and 8.85% from 5.29 to 5.78 GHz. The measured (simulated) |S11| shows four resonant frequencies at 3.68, 3.89, 5.39 and 5.69 GHz (3.74, 3.96, 5.43 and 5.74 GHz). The measured (simulated) maximum realized gains in the two bands are 6.04 (6.93) dBi and 6.64 (7.59) dBi, respectively. Furthermore, the measured (simulated) radiation nulls are observed at 3.38, 4.87 and 6.74 GHz (3.44, 4.89 and 6.9 GHz), verifying the dual-band filtering response.

#### 4.1.3. Radiation Pattern Analysis

**Figure 17:**
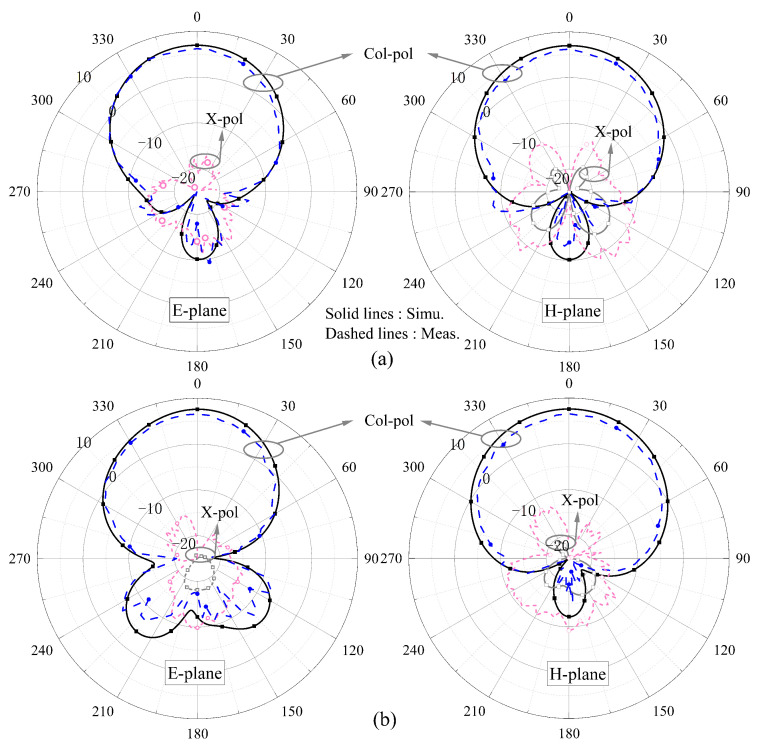
Measured (simulated) radiation patterns at (**a**) 3.82 (3.87) GHz and (**b**) 5.54 (5.58) GHz.

Figure 17 presents the measured (simulated) normalized radiation patterns at 3.82 (3.87) GHz and 5.54 (5.58) GHz, indicating a favorable front-to-back ratio. In Figure 17a, the simulated cross-polarization is less than −25 dB, so it does not appear.

#### 4.1.4. Comparison with Filtering Antennas

Table 3 presents a comparison between the proposed antenna and previously reported filtering antennas. It can be observed that the proposed antenna has the advantage of dual-band and dual-bandpass filtering response compared to [4,8,25,26]. Compared to [11,13], the proposed antenna features a simpler design and lower cost. Additionally, the proposed antenna exhibits consistent radiation characteristics.

### 4.2. Two-Element MIMO Array I Results and Comparison

#### 4.2.1. Fabricated Prototype of Two-Element MIMO Array I

**Figure 18:**
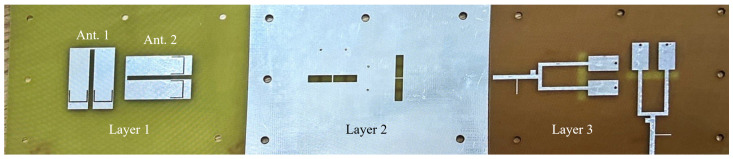
Fabricated prototype of the two-element MIMO array I.

The Two-Element MIMO Array I has been fabricated and evaluated. The prototype is depicted in Figure 18. Layer 1 consists of two vertically placed U-shaped patches with U-slots, Layer 2 is a ground plane etched with four rectangular slots and four circular grooves and Layer 3 features two vertically placed feedlines.

#### 4.2.2. S-Parameters and Realized Gain Analysis

**Figure 19:**
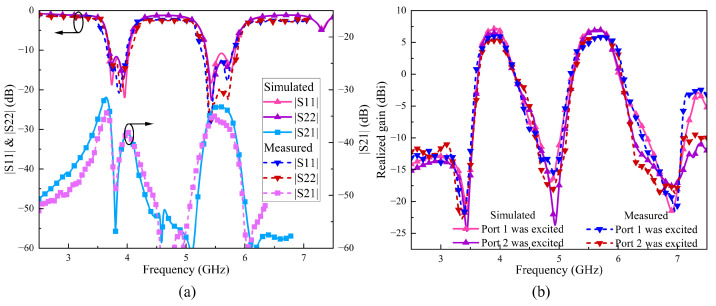
The measured and simulated (**a**) S-parameters and (**b**) realized gain of the two-element MIMO array I.

As Figure 19a illustrates, the measured −10 dB impedance bandwidth of Ant. 1 is 8.88% in the range of 3.66 to 4.00 GHz and 9.76% in the range of 5.26 to 5.80 GHz. The measured −10 dB impedance bandwidth of Ant. 2 is 8.76% from 3.71 to 4.05 GHz and 9.89% from 5.22 to 5.76 GHz. The measured |S21| is less than −34.37 and −34.97 dB within the passband, representing an isolation improvement of 12.77 and 10.17 dB compared to Ref. arrays 3 and 4, respectively. The measured results at 3.81 GHz show the expected decoupling null, achieving a maximum isolation of −48.71 dB. Moreover, in Figure 19b, the measured (simulated) realized gain reveals that both Ant. 1 and Ant. 2 exhibit excellent dual-band filtering response, with each antenna element showing three radiation nulls. The measured (simulated) maximum realized gain for Ant. 1 in the dual-band are 5.89 (7.19) dBi and 5.83 (6.99) dBi, while those for Ant. 2 are 5.29 (6.49) dBi and 5.86 (6.97) dBi.

#### 4.2.3. Radiation Pattern Analysis

**Figure 20:**
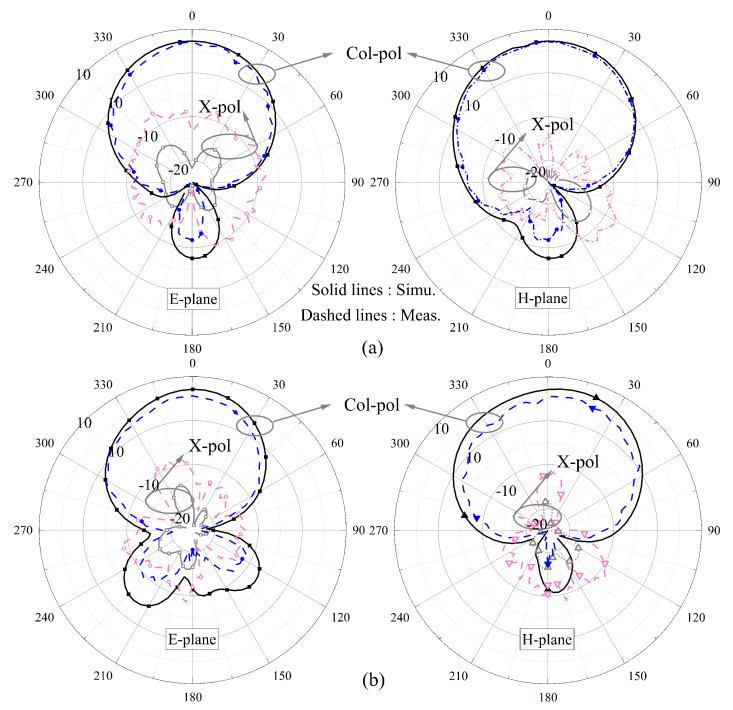
Measured (simulated) radiation patterns at (**a**) 3.83 (3.88) GHz and (**b**) 5.53 (5.59) GHz of the two-element MIMO array I when port 1 is excited and port 2 is loaded.

Figure 20 presents the measured (simulated) normalized radiation patterns when port 1 is excited and port 2 is loaded at 3.83 (3.88) GHz and 5.53 (5.59) GHz, indicating consistent broadside radiation patterns. The results indicate that the radiation patterns are almost unaffected.

### 4.3. Two-Element MIMO Array II Results and Comparison

#### 4.3.1. Fabricated Prototype of Two-Element MIMO Array II

**Figure 21:**
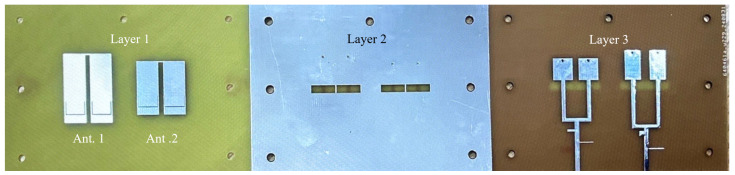
Fabricated prototype of the two-element MIMO array II.

The prototype of the two-element MIMO array II is shown in Figure 21. Layer 1 consists of two horizontally placed U-shaped patches with U-slots, Layer 2 features a ground plane etched with four horizontal rectangular slots and four circular grooves and Layer 3 contains two horizontally placed feedlines.

#### 4.3.2. S-Parameters and Realized Gain Analysis

**Figure 22:**
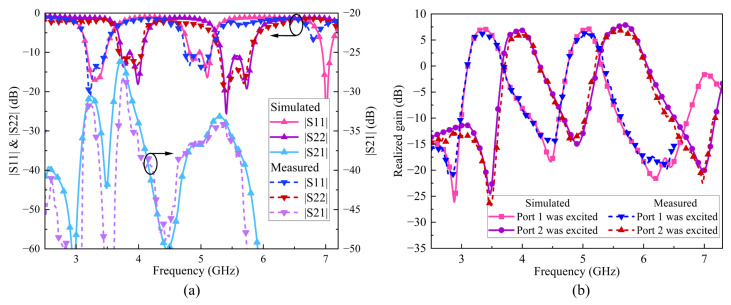
The measured and simulated (**a**) S-parameters and (**b**) realized gain of the two-element MIMO array II.

Figure 22a presents the measured and simulated S-parameters. The measured −10 dB impedance bandwidth of Ant. 1 is 9.15% within the range of 3.13 to 3.43 GHz and 8.32% within 4.72 to 5.13 GHz. For Ant. 2, the measured −10 dB impedance bandwidth is 9.65% within 3.67 to 4.00 GHz and 8.32% within 5.29 to 5.77 GHz. Across the four bands, the |S21| is less than −31.76, −28.14, −37.25 and −34.64 dB, respectively, achieving isolation improvements of at least 15.56 dB, 10.24 dB, 17.15 dB and 13.44 dB compared to Ref. arrays 5 and 6. Additionally, the measured and simulated boresight gain curves of the binary MIMO array 2 are plotted in Figure 22b. In the four bands, the measured (simulated) max realizes gains are 6.19 (7.11) dBi, 5.99 (6.89) dBi, 6.27 (7.22) dBi and 6.80 (7.88) dBi, demonstrating excellent filtering performance.

#### 4.3.3. Radiation Pattern Analysis

**Figure 23:**
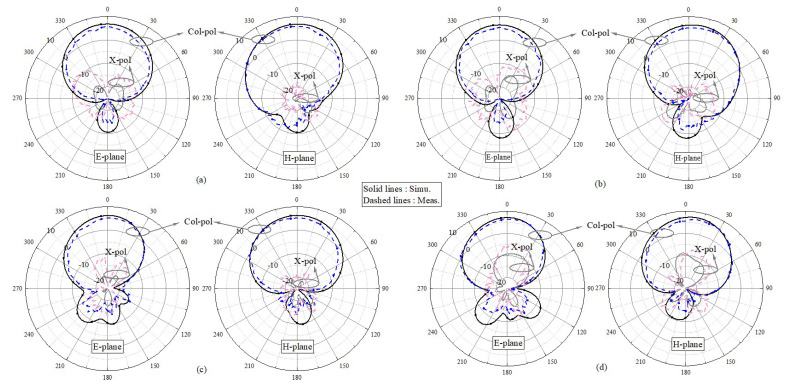
Measured (simulated) radiation patterns at (**a**) 3.28 (3.32) GHz and (**b**) 4.93 (4.99) GHz when port 1 was excited and (**c**) 3.84 (3.89) GHz and (**d**) 5.53 (5.57) GHz when port 2 was excited.

The measured and simulated normalized radiation patterns of Ant. 1 and 2 are shown Figure 23; even without any decoupling structures, the radiation patterns do not deteriorate and the measured results agree well with the simulations.

#### 4.3.4. Comparison with MIMO Arrays

Table 4 provides a detailed comparison with previously reported single-band and dual-band decoupling methods. By contrast, this study mainly demonstrates three advantages:

(1) The decoupling method is simple. High isolation can be achieved using aperture coupling characteristics and filtering effects.
(2) It facilitates multi-band decoupling. Compared to [25,26], only two elements are needed for a MIMO array operating in four bands.
(3) It achieves good filtering response and radiation performance at a low cost.

### 4.4. Error Analysis

**Figure 24:**
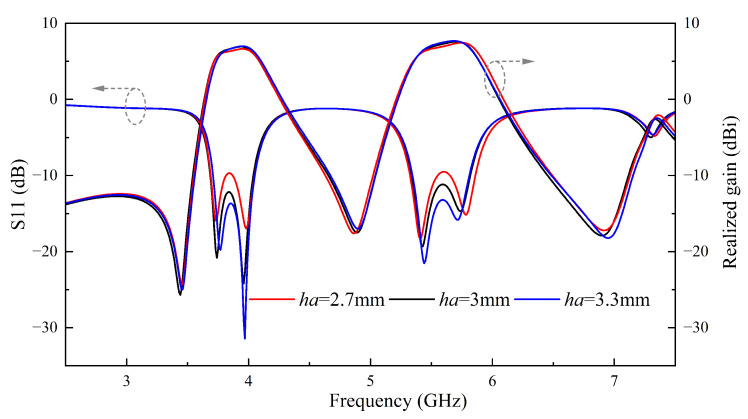
Performance variation of antennas at different heights of air layers.

From Figure 17, Figure 20 and Figure 23, it is evident that the co-polarization in the measured radiation patterns shows a good alignment. However, the level of cross-polarization is excessively high, primarily due to the strong y-directed current caused by the etched two U-slots on the patch [31,32]. To effectively suppress the high cross-polarization levels, differential feeding can be considered for further improvement [12]. The height of the air layer also influences the measurement results. As shown in Figure 24, with the increase in height, the impedance matching gradually improves and the gain slightly increases, while the bandwidth slightly decreases. To achieve a good bandwidth, realized gain characteristics and a low profile, we ultimately select a 3 mm air layer. Therefore, the fabricated antenna elements can be improved in measurements by fine-tuning the height of the nylon nuts. But measured gain is slightly reduced compared to the simulated values. Furthermore, the discrepancies between simulation and measurement results are related to dielectric loss, conductor loss, transmission cables, testing environment and fabrication errors.

## 5. Conclusions

This paper presents a novel dual-band filtering antenna that integrates self-decoupling and filtering functions. On the one hand, the short-circuited SIR structure, U-shaped slots and an open stub collectively generate three radiation nulls, providing the antenna with excellent dual-band filtering response. On the other hand, these structures introduce two additional resonant modes that combine with the TM10 and TM20 modes of the patch, forming dual-passband characteristics. And aperture coupling imparts inherent self-decoupling characteristics to vertically placed antenna elements, effectively suppressing near-field coupling with adjacent elements. As a result, the antenna’s self-decoupling and filtering properties make it an ideal candidate for arrays operating in dual- and quad-bands. This dual-band filtering antenna element boasts advantages of simple design and low cost, enabling enhanced isolation in multi-band MIMO arrays without the addition of any decoupling structures. The measurement results confirm this. We believe this patch antenna will be widely applicable in multi-antenna systems operating in multiple frequency bands, particularly in systems comprising closely spaced dual-band filtering antenna elements operating in the same or adjacent dual-frequency bands, where it demonstrates excellent performance.

This paper focuses on the design of dual-band and quad-band high-isolation dual antenna arrays utilizing linear polarized antenna elements. As a significant direction for future work, we aim to develop a large MIMO multi-antenna system capable of operating across multiple frequency bands to facilitate functions such as multi-band beam control and information processing. However, the proposed multi-layer dielectric substrates and air layers limit its applicability in millimeter-wave and chip-based systems. To address this, we consider adopting a multi-metal layer approach to better align with the requirements of modern communication systems [33].

## Figures and Tables

**Table 1:** Specific parameters of the antenna element 1.

Parameter	Value	Parameter	Value	Parameter	Value
$D_{1}$	2 mm	$F_{L4}$	3 mm	$P_{L}$	21 mm
$D_{2}$	0.5 mm	$F_{W1}$	8 mm	$P_{W}$	8.5 mm
$D_{3}$	8 mm	$F_{W2}$	1.5 mm	$R_{1}$	0.5 mm
$D_{4}$	3 mm	$F_{W3}$	0.4 mm	$S_{L1}$	8 mm
$F_{L1}$	8.8 mm	$F_{W4}$	1 mm	$S_{L2}$	4.2 mm
$F_{L2}$	15 mm	$G_{L}$	70 mm	*W*	3 mm
$F_{L3}$	5.8 mm	*L*	10 mm	$W_{S}$	0.2 mm

**Table 2:** Specific parameters of the antenna element 2.

Parameter	Value	Parameter	Value	Parameter	Value
$D_{1}$	2 mm	$F_{L4}$	2 mm	$P_{L}$	26.6 mm
$D_{2}$	0.5 mm	$F_{W1}$	6.5 mm	$P_{W}$	8.5 mm
$D_{3}$	8 mm	$F_{W2}$	1.5 mm	$R_{1}$	0.5 mm
$D_{4}$	1 mm	$F_{W3}$	0.4 mm	$S_{L1}$	8 mm
$F_{L1}$	11.8 mm	$F_{W4}$	2 mm	$S_{L2}$	4.9 mm
$F_{L2}$	18 mm	$G_{L}$	70 mm	*W*	3.2 mm
$F_{L3}$	6.5 mm	*L*	10 mm	$W_{S}$	0.5 mm

**Table 3:** Comparison of the filtering antennas.

	Single/Dual-Band Operation	Height	Design Complexity	Filtering Response	Filtering Method	Substrate (Cost)
[4]	Single	6.6 mm	Complex	Yes	additional coupling between source/load and nonadjacent resonators	RO4003C (High)
[8]	Single	13 mm	Moderate	Yes	Shorting pins & U-slot & stacked patch	er = 2.65 thickness = 2.3 mm
[11]	Dual	0.79 mm	Complex	Yes	Dual-mode T-shaped resonator & two vertically placed patches	RO4003C (High)
[13]	Dual	3.623 mm	moderate	Yes	Integrate a dual-mode resonator in U-slot patch	RO4003C (High)
[25]	Single	14 mm	Simple	Yes	Shorting pins & E-slot & stacked patch	er = 2.65 thickness = 3 mm
[26]	Single	1.575 mm	Complex	Yes	A secondary stub-loaded inverted-F radiator	RO5880 (High)
This work	Dual	5 mm	Simple	Yes	SIR & U-slots & an open stub	FR4 (Low)

**Table 4:** Comparison of the MIMO arrays.

	Single/Dual-Band Decoupling	Isolation/ Improvement (dB)	Edge-to-Edge Distance (λ0)	Decoupling Method	Filtering Response	Design Complexity
[16]	single-band	33.6/18.9	0.13	Filtering network	Yes	Complex
[19]	single-band	27/20	0.03	Modes Cancellation	No	Complex
[23]	Single	18.7/11	0.06	symmetric meander-line slots	No	moderate
[25]	Dual-band	30/8	0.25	Filtering function	Yes	Simple
[26]	Single-band	16.7/9.7	0.054	Inverted-F radiator	Yes	Complex
	Dual-band	16.9/7.5	0.056	Filtering function		
[27]	Dual	26/15	0.039	hybrid coupling interface technique	No	Moderate
This work	Dual-band	34.37/10.17	0.086	Aperture-coupling	Yes	Simple
	Squad-band	28.14/10.24	0.11	Filtering function		

## Data Availability

Data is contained within the article.

## Abbreviations

MIMO	Multiple-Input Multiple-Output
SIR	Stepped-Impedance-Resonator
RLC	Resistor-Inductor-Capacitor
SSIRs	Shared Stepped Impedance Resonators
CSRRs	Complementary Split-Ring Resonators

## References

[1] Xiang K.R., Chen F.C., Tan Q., Chu Q.X. (2021). High-Selectivity Filtering Patch Antennas Based on MultiPath Coupling Structures. IEEE Trans. Microw. Theory Tech..

[2] Qian J.F., Chen F.C., Ding Y.H., Hu H.T., Chu Q.X. (2019). A Wide Stopband Filtering Patch Antenna and its Application in MIMO System. IEEE Trans. Antennas Propag..

[3] Hu H.T., Chen F.C., Qian J.F., Chu Q.X. (2017). A Differential Filtering Microstrip Antenna Array With Intrinsic Common-Mode Rejection. IEEE Trans. Antennas Propag..

[4] Zhang B., Xue Q. (2018). Filtering Antenna With High Selectivity Using Multiple Coupling Paths From Source/Load to Resonators. IEEE Trans. Antennas Propag..

[5] Chen X., Zhao F., Yan L., Zhang W. (2013). A Compact Filtering Antenna With Flat Gain Response Within the Passband. IEEE Antennas Wirel. Propag. Lett..

[6] Liu S., Wang Z., Dong Y. (2023). A Compact Filtering Patch Antenna With High Suppression Level and Its CP Application. IEEE Antennas Wirel. Propag. Lett..

[7] Wei F., Liu X., Ding X.Z., Zhao X.B., Qin P.Y. (2023). A Balanced Filtering Antenna Array With High Gain, Steep Selectivity, and Multiradiation Nulls Parallel-Fed by Differential Broadband Network. IEEE Trans. Antennas Propag..

[8] Zhang X.Y., Duan W., Pan Y.M. (2015). High-Gain Filtering Patch Antenna Without Extra Circuit. IEEE Trans. Antennas Propag..

[9] Hu K.Z., Huang H.Y., Tang M.C., Chen Z., Yan D., Wang P. (2023). A Single-Layer Wideband Differential-Fed Filtering Antenna With High Selectivity. IEEE Trans. Antennas Propag..

[10] Hsieh C.Y., Wu C.H., Ma T.G. (2015). A Compact Dual-Band Filtering Patch Antenna Using Step Impedance Resonators. IEEE Antennas Wirel. Propag. Lett..

[11] Dhwaj K., Jiang L.J., Itoh T. (2018). Dual-Band Filtering Antenna With Novel Transmission Zero Characteristics. IEEE Antennas Wirel. Propag. Lett..

[12] Li D., Tang M.C., Wang Y., Hu K.Z., Ziolkowski R.W. (2022). Dual-Band, Differentially-Fed Filtenna With Wide Bandwidth, High Selectivity and Low Cross-Polarization. IEEE Trans. Antennas Propag..

[13] Mao C.X., Gao S., Wang Y., Sanz-Izquierdo B., Wang Z., Qin F., Chu Q.X., Li J., Wei G., Xu J. (2016). Dual-Band Patch Antenna With Filtering Performance and Harmonic Suppression. IEEE Trans. Antennas Propag..

[14] Liu X., Sanz-Izquierdo B., Zhang H., Gao S., Hu W., Yang X.X., Sri Sumantyo J.T. (2024). Differentially Fed Dual-Band Base Station Antenna With Multimode Resonance and High Selectivity for 5G Applications. IEEE Trans. Antennas Propag..

[15] Larsson E.G., Edfors O., Tufvesson F., Marzetta T.L. (2014). Massive MIMO for next generation wireless systems. IEEE Commun. Mag..

[16] Qian J., Sanz Izquierdo B., Gao S., Wang H., Zhou H., Xu H. (2023). A Cascaded Resonator Decoupling Network for Two Filtering Antennas. IEEE Antennas Wirel. Propag. Lett..

[17] Zou X.J., Wang G.M., Wang Y.W., Li H.P. (2019). An Efficient Decoupling Network Between Feeding Points for Multielement Linear Arrays. IEEE Trans. Antennas Propag..

[18] Yuan H., Chen F.C. (2023). A Mixed Decoupling Scheme Based on AMC and ADS for Dual-Polarized Antenna Array. IEEE Trans. Antennas Propag..

[19] Guo C., Jia Y., Wang Y., Zhai H. (2023). A Dual-Polarized Antenna Array With High Port Isolation Through TM03/04 Modes Cancellation. IEEE Antennas Wirel. Propag. Lett..

[20] Lai Q.X., Pan Y.M., Zheng S.Y. (2022). Mode-Counteraction Based Self-Decoupling in Circularly Polarized MIMO Microstrip Patch Array. IEEE Trans. Antennas Propag..

[21] Qian J., Izquierdo B.S., Gao S., Wang H., Zhou H., Xu H. (2024). A Novel Low-Cost H-Plane Decoupling Technique for Two Closely Placed Patch Antennas Using Electric and Magnetic Coupling Cancellation. IEEE Trans. Antennas Propag..

[22] Yang W., Chen L., Pan S., Che W., Xue Q. (2022). Novel Decoupling Method Based on Coupling Energy Cancellation and its Application in 5G Dual-Polarized High-Isolation Antenna Array. IEEE Trans. Antennas Propag..

[23] Hwangbo S., Yang H.Y., Yoon Y.K. (2017). Mutual Coupling Reduction Using Micromachined Complementary Meander-Line Slots for a Patch Array Antenna. IEEE Antennas Wirel. Propag. Lett..

[24] Fu S., Wang Y., Li C., Xu Z. (2024). Dual-Circularly Polarized STAR Antenna Array With High Realized Gain and Enhanced Isolation. IEEE Antennas Wirel. Propag. Lett..

[25] Zhang Y., Zhang X.Y., Ye L.H., Pan Y.M. (2016). Dual-Band Base Station Array Using Filtering Antenna Elements for Mutual Coupling Suppression. IEEE Trans. Antennas Propag..

[26] Li M., Tian S., Tang M.C., Zhu L. (2022). A Compact Low-Profile Hybrid-Mode Patch Antenna With Intrinsically Combined Self-Decoupling and Filtering Properties. IEEE Trans. Antennas Propag..

[27] Deng H., Zhu L. (2023). Self-Decoupled Dual-Frequency Patch Antennas via Hybrid Coupling Interface Technique. IEEE Antennas Wirel. Propag. Lett..

[28] Liu S., Wang Z., Dong Y. (2023). A Compact Coupling-Fed Patch Antenna With Quasi-Elliptic Filtering Response. IEEE Antennas Wirel. Propag. Lett..

[29] Lu Y.J., Chen S.Y., Hsu P. (2012). A Differential-Mode Wideband Bandpass Filter With Enhanced Common-Mode Suppression Using Slotline Resonator. IEEE Microw. Wirel. Compon. Lett..

[30] Wei F., Zhao X.B., Shi X.W. (2019). A Balanced Filtering Quasi-Yagi Antenna With Low Cross-Polarization Levels and High Common-Mode Suppression. IEEE Access.

[31] Tian M., Yan N., Luo Y., Ma K. (2021). A Low-Cost High-Gain Filtering Patch Antenna Using SISL Technology for 5G Application. IEEE Antennas Wirel. Propag. Lett..

[32] Liu Y.T., Leung K.W., Yang N. (2020). Compact Absorptive Filtering Patch Antenna. IEEE Trans. Antennas Propag..

[33] Alibakhshikenari M., Virdee B.S., Khalily M., See C.H., Abd-Alhameed R., Falcone F., Denidni T.A., Limiti E. (2020). High-Gain On-Chip Antenna Design on Silicon Layer With Aperture Excitation for Terahertz Applications. IEEE Antennas Wirel. Propag. Lett..

